# Recombinant Human Thyrotropin-Aided Radioiodine Therapy in Tracheal Obstruction by an Invading Well-Differentiated Thyroid Carcinoma

**DOI:** 10.1155/2013/579527

**Published:** 2013-02-27

**Authors:** Nickolaos Pontikides, Spyridon Karras, Antonios Papagiannis, Athina Kaprara, Panagiotis Anagnostis, George Noussios, Argyrios Doumas, Apostolos Goropoulos, Ioannis Iakovou, Georgios Kotronis, Konstantinos Bantis, Gerasimos Krassas

**Affiliations:** ^1^Department of Endocrinology-Diabetes, Panagia General Hospital, 22 Plastira Street, 55132 Thessaloniki, Greece; ^2^Pulmonary Department, St. Luke's Hospital, 55236 Thessaloniki, Greece; ^3^Department of Endocrinology, Hippokration Hospital of Thessaloniki, 54642 Thessaloniki, Greece; ^4^Laboratory of Anatomy Department of Physical Education and Sports Medicine, Aristotle University of Thessaloniki, 54636 Thessaloniki, Greece; ^5^Third Department of Nuclear Medicine, Papageorgiou Hospital, Aristotle University of Thessaloniki, 56429 Thessaloniki, Greece; ^6^Department of Endocrine Surgery, St. Luke's Hospital, 55236 Thessaloniki, Greece; ^7^3rd Department of Medicine, Papageorgiou Hospital, Aristotle University of Thessaloniki, 56429 Thessaloniki, Greece

## Abstract

Papillary thyroid carcinomas (PTCs) usually extend to lymph nodes in the neck and mediastinum. Rarely, they invade the neighboring upper airway anatomical structures. We report a 56-year-old woman who presented with symptoms of upper airway obstruction. Imaging studies revealed a lesion derived from the thyroid which invaded and obstructed the trachea, which appeared to be a highly differentiated PTC. Total thyroidectomy was performed, with removal of the endotracheal part of the mass along with the corresponding anterior tracheal rings. Two months later, a whole body I^131^ scan after recombinant human thyroid-stimulating hormone (rh-TSH) administration was performed and revealed a residual mass in upper left thyroid lobe. Subsequently, 150 mCi I^131^ were given following rh-TSH administration. Nine months later, there was no sign of residual tumor. This case is the first one reported in the literature regarding rh-TSH administration prior to RAI ablation in a PTC obstructing the trachea.

## 1. Introduction

Papillary thyroid carcinoma (PTC) represents approximately 75% of all thyroid cancers [1]. Their extrathyroidal extension often involves regional lymph nodes in the neck and mediastinum [1]. It is relatively rare that a PTC invades the upper airway anatomical structures or the digestive tract [2]. Therefore, invasion of the trachea by a PTC could mimic other common clinical conditions, and this could result in pitfalls in the diagnosis and management of this extraordinary manifestation. This rare entity requires a high index of clinical suspicion and a multidisciplinary approach by a group of specialists in order to provide an effective therapeutic outcome.

We report a rare case of PTC invading the trachea who presented with symptoms of upper airway obstruction (UAO) and we describe our therapeutic approach.

## 2. Case Report

A 56-year-old woman was admitted to the Pulmonary Outpatient Clinic complaining of fluctuating cough and dyspnea. She had been under immunotherapy for 2 years due to allergic asthma, and, spontaneously, she complained of sore throat and mild haemoptysis. Clinical examination revealed scattered wheezes from the lungs without any abnormal findings from the pharynx. Spirometry showed a typical obstructive pattern, attributed to asthma. Her medical history was remarkable of subclinical hypothyroidism, adequately treated with levothyroxine.

The patient was lost to follow-up and returned 3 years later with increased dyspnea, neck discomfort, deepening voice, and wheezing. On examination, she had prolonged expiration with wheezing and worsening in the spirometric parameters. Despite oral corticosteroid administration, the symptoms gradually deteriorated and she developed inspiratory stridor and severe dyspnea with spirometric evidence of severe upper airway obstruction. She was admitted to the Intensive Care Unit for further investigation and management.

**Figure 1:**
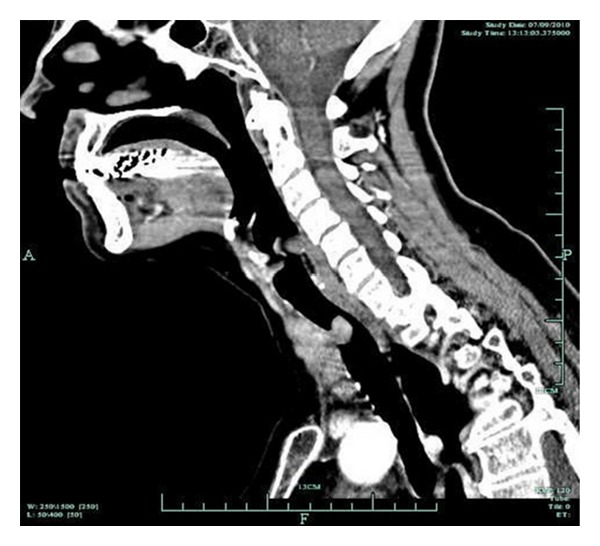
CT scan of the neck and mediastinum which revealed a mass originating from the thyroid gland, invading and severely obstructing the lumen of the trachea.

**Figure 2:**
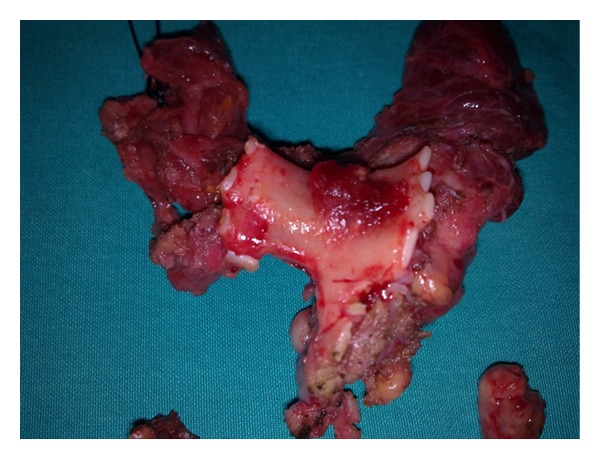
Macroscopic appearance of the mass along with the corresponding anterior portions of three tracheal rings.

Computed tomography (CT) scan of the neck and mediastinum revealed an abnormal lesion (3.5 × 2.8 × 3.7 cm), originating from the right lobe of the thyroid gland, invading and severely obstructing the lumen of the trachea (Figure 1). No lymph nodes or other abnormalities were observed. In view of severe dyspnea, a low tracheostomy was performed. An open biopsy from an unstable mass penetrating the anterolateral wall of the trachea and from the endotracheal mass by endoscopy was obtained. The biopsies were compatible of highly differentiated PTC with additional extensive inflammatory lesions and necrotic hemorrhagic material. Eventually, the patient underwent a total thyroidectomy and resection of the endotracheal part of the mass along with the corresponding anterior portions of 3 tracheal rings (Figure 2). To maintain the patency of the airway, a Montgomery T-tube was placed. The patient was easily extubated, and breathing and speech were fully restored. The histological examination of the surgical material confirmed the presence of highly differentiated PTC of the isthmus, stage IVa, with local extension to the pretracheal fibromuscular tissues and extensive invasion of the tracheal cartilage. The patient recovered with no complications.

Two months later, a whole body I^131^ scan (WBS) after recombinant human thyroid-stimulating hormone (rh-TSH) administration was performed, which demonstrated a residual mass in upper left thyroid lobe. Subsequently, 150 mCi I^131^ were given after administration of rh-TSH again. Nine months later, a WBS showed no residual tumor and Tg levels after rh-TSH were 1.1 ng/mL. The physical status of the patient was excellent thereafter.

## 3. Discussion

This case demonstrates an uncommon clinical manifestation of a well-differentiated PTC. Our patient presented with severe UAO due to involvement of the trachea by a primary thyroid tumor. Following endoscopic biopsy and histological confirmation of PTC, the tumor was successfully removed, followed by radioiodine ablation after rh-TSH administration. To our knowledge, this is the first case describing rh-TSH administration prior to radioiodine ablation for a PTC obstructing the trachea.

**Figure 3:**
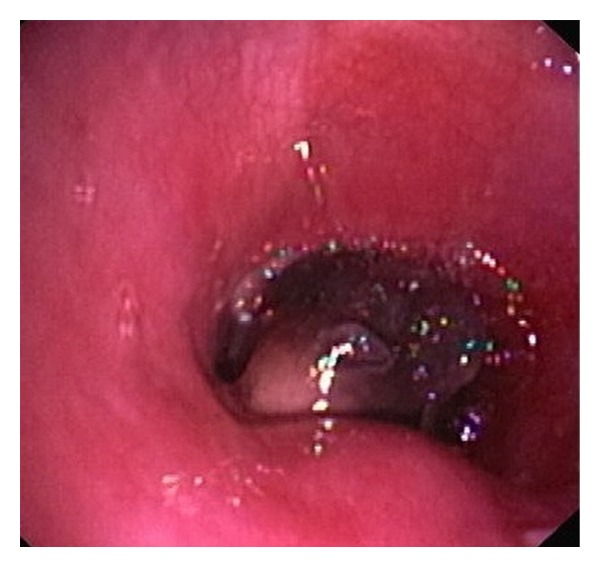
The bronchoscopic image revealed the extensive intraluminal growth of the mass.

Approximately 7–10% of all thyroid cancers invade the larynx or trachea. UAO may occur in up to 25% of cases of undifferentiated anaplastic thyroid carcinoma. In contrast, PTC frequently metastasizes to the regional lymph nodes but uncommonly can extend to adjacent organs [2]. Invasion of the trachea by a PTC is very uncommon, although several cases have been reported in children and adults [3–6]. In our case, apart from tracheal wall diffusion, extensive intraluminal growth developed (Figure 3), which is an extremely rare manifestation of PTC. On a pathological basis, it is also interesting that a soft tissue tumor can erode the tracheal cartilage and gradually cause intratracheal lumen obstruction. Finally, upper airway obstruction can be a life-threatening complication of extended PTC if not diagnosed and treated in time [3–6].

A high index of clinical suspicion is necessary, since symptoms may be subtle and patients may not be referred to specialists. In most previous reports, respiratory symptoms, such as hemoptysis, dyspnea, wheezing, and persistent cough, were common, although in some cases acute airway obstruction was the first manifestation [3–6]. The differential diagnosis of UAO includes serious anaphylactic reactions, chemical respiratory burns, infections such as croup, epiglottitis, and other viral or bacterial diseases, as well as peritonsillar and retropharyngeal abscesses, throat cancer, and foreign bodies. Thyroid cancer, although rare as a cause of UAO, should also be considered [7]. In our case, there was no medical history, or other evidence, suggestive of PTC. Symptoms of airway obstructive disease had existed several years before and gradually increased during the last 3 years. These symptoms were initially attributed to asthma and were managed accordingly; the partial relief of the obstruction with medical therapy led to a delay in diagnosis.

Physical examination may reveal a solitary, firm thyroid nodule and/or cervical lymph nodes of the neck, assisted by Imaging including plain X-rays of the chest, CT of the neck and upper mediastinum, and thyroid gland ultrasonography (US). US can be used to assess lesions even as small as 2 mm, distinguishing solid from cystic ones. CT and magnetic resonance imaging (MRI) are indicated for tumors greater than 3 cm that may extend to other structures, such as the mediastinum, the retropharyngeal region, and the aerodigestive tract [8]. Invasion of the trachea will be readily visible with these modalities and can be confirmed by naso-tracheoscopy, which also allows biopsy [9].

At this point, two main issues arise. The first is to diagnose the exact nature of the invading mass which will determine further treatment; the other is to prevent acute respiratory complications resulting from UAO. In the presence of thyroid nodules, previously visible on thyroid US, cytological examination of material taken with fine needle aspiration can be helpful in the diagnostic procedure [10]. In our case, in the absence of a relevant medical history of endocrine related cancer, our approach was directed towards the identification of the tumor origin.

According to previous data [9], thyroid cancer usually invades the trachea between the cartilage rings. Mucosal redness and localized mucosal changes are endoscopical signs of tracheal invasion [9]. Preoperative endoscopic assessment of trachea invasion is also very important for determining the appropriate treatment for thyroid cancer invading the tracheal wall. CT and MRI are useful when there is large intraluminal involvement of cancer, but they are not good enough for determining the depth and length of tracheal invasion [9].

The appropriate management of such patients should include radical resection of the tumor and subsequent tracheal reconstruction. The procedures that are mainly performed are circular resection, end-to-end anastomosis, and window resection. If the invasion is limited to the surface layer of the tracheal wall, it can be treated through a procedure known as “shaving” [11], but this technique may not result in complete resection and recurrence is common [12]. When the tumor extends to the lumen of the trachea, then all the layers of the trachea should be resected. If, as in our case, only the lateral or anterior wall is involved, a window of the involved trachea with normal margins is resected *en bloc* with the tumor [13]. If the tumor causes a near-circumferential involvement of the tracheal wall, a wide resection of the trachea is required; circular resection and end-to-end anastomosis are performed. The maximum length of tracheal resection that allows end-to-end anastomosis is equivalent to 7 rings of the trachea or a longitudinal axis of 5-6 cm [11]. However, it has been reported that an end-to-end anastomosis is possible for defects up to 7 cm long [14]. Complete resection of laryngotracheal invasion is followed by the longest overall and disease-free survival [15]. In our patient, an initial low tracheostomy was performed, and immediately after histological confirmation of the tumor origin, the patient underwent total thyroidectomy including resection of the endotracheal part of the mass along with the involved tracheal cartilage.

Postoperative ablation with RAI is mandatory in order to minimize the risk of recurrence in these aggressive thyroid carcinomas. Therefore, appropriate doses of RAI should be administered in keeping with current guidelines [1]. In cases of a visible uptake after whole body scan, RAI administration should be repeated.

Rh-TSH stimulation is a useful tool in the postoperative management of differentiated thyroid carcinomas in preparation for Tg testing with or without radioiodine imaging for detection of thyroid remnants of well-differentiated PTC in adult postthyroidectomy patients maintained on hormone suppression therapy. Low-risk patients with well-differentiated PTC who have undetectable serum Tg levels on thyroid hormone suppression could be followed by assaying rh-TSH stimulated serum Tg levels [1]. Current guidelines support that rh-TSH preparation for RAI therapy in patients with distant metastases can be considered in patients with underlying comorbidities that make iatrogenic hypothyroidism potentially risky, such as in our case [1].

In conclusion, we described up a case report of trachea obstruction due to invasion of a highly differentiated PTC. Total thyroidectomy with endotracheal part of the metastatic lesion along with the corresponding anterior tracheal rings was performed, followed by RAI ablation after rh-TSH administration. There was no sign of residual tumor or disease recurrence during follow-up. We conclude that rh-TSH can be used as preparation for RAI therapy in patients with metastases who cannot tolerate the iatrogenic hypothyroidism.
